# Quantifying Antimicrobial Resistance at Veal Calf Farms

**DOI:** 10.1371/journal.pone.0044831

**Published:** 2012-09-07

**Authors:** Angela B. Bosman, Jaap Wagenaar, Arjan Stegeman, Hans Vernooij, Dik Mevius

**Affiliations:** 1 Department of Infectious Diseases and Immunology, Faculty of Veterinary Medicine, Utrecht University, Utrecht, The Netherlands; 2 Central Veterinary Institute of Wageningen UR, Lelystad, The Netherlands; 3 Department of Farm Animal Health, Faculty of Veterinary Medicine, Utrecht University, Utrecht, The Netherlands; Washington State University, United States of Amercia

## Abstract

This study was performed to determine a sampling strategy to quantify the prevalence of antimicrobial resistance on veal calf farms, based on the variation in antimicrobial resistance within and between calves on five farms. Faecal samples from 50 healthy calves (10 calves/farm) were collected. From each individual sample and one pooled faecal sample per farm, 90 selected *Escherichia coli* isolates were tested for their resistance against 25 mg/L amoxicillin, 25 mg/L tetracycline, 0.5 mg/L cefotaxime, 0.125 mg/L ciprofloxacin and 8/152 mg/L trimethoprim/sulfamethoxazole (tmp/s) by replica plating. From each faecal sample another 10 selected *E. coli* isolates were tested for their resistance by broth microdilution as a reference. Logistic regression analysis was performed to compare the odds of testing an isolate resistant between both test methods (replica plating vs. broth microdilution) and to evaluate the effect of pooling faecal samples. Bootstrap analysis was used to investigate the precision of the estimated prevalence of resistance to each antimicrobial obtained by several simulated sampling strategies. Replica plating showed similar odds of *E. coli* isolates tested resistant compared to broth microdilution, except for ciprofloxacin (OR 0.29, p≤0.05). Pooled samples showed in general lower odds of an isolate being resistant compared to individual samples, although these differences were not significant. Bootstrap analysis showed that within each antimicrobial the various compositions of a pooled sample provided consistent estimates for the mean proportion of resistant isolates. Sampling strategies should be based on the variation in resistance among isolates within faecal samples and between faecal samples, which may vary by antimicrobial. In our study, the optimal sampling strategy from the perspective of precision of the estimated levels of resistance and practicality consists of a pooled faecal sample from 20 individual animals, of which 90 isolates are tested for their susceptibility by replica plating.

## Introduction

Antimicrobial resistance is an increasing problem and a global threat for human and animal health. Antimicrobial usage is considered a major determinant for emergence, selection and dissemination of antimicrobial resistant microorganisms [1], [2], [3]. Moreover, there is increasing evidence that transmission of antimicrobial drug resistant commensal and zoonotic bacteria, like methicillin resistant *Staphylococcus aureus* (MRSA) and extended spectrum beta-lactamase producing *Escherichia coli*, from food producing animals to humans may occur [4], [5], [6], [7], [8]. In response to public and animal health concerns, antimicrobial resistance monitoring programmes have been developed and implemented on national [9] and European level [10]. These programmes monitor and report antimicrobial drug resistance among commensal and zoonotic bacteria from food producing animals, like veal calves. These monitoring programmes, however, provide no information on the prevalence of antimicrobial resistance on farm-level while this information is vital to identify risk factors for the development and spread of antimicrobial resistance within farms.

Studies to determine the prevalence of antimicrobial resistance on farms are generally based upon measuring resistance of commensal bacteria from the intestines of farm animals, like *Enterococcus* spp. and *E. coli.* These bacteria are considered good indicator organisms to monitor the effects of the selective pressure exerted by the use of antimicrobials in food animals [11]. Moreover, *E. coli* and enterococci constitute a natural part of the intestinal flora of animals, which facilitates the comparison of resistance percentages between populations [12], [13].

Bacterial isolates can be tested for their resistance in several ways, for example using broth microdilution or replica plating. With broth microdilution an isolate is tested against a panel of antimicrobials. This method is used worldwide in monitoring programmes and can be considered to be the ‘gold standard’ [13]. Replica plating on the other hand is a more feasible and cost-effective method to quantify resistance within animal populations since multiple isolates can be tested simultaneously for their resistance using agar plates containing an antimicrobial at a breakpoint concentration [14], [15].

In order to determine resistance on herd-level a multi-level approach is needed [16]. One needs to know how many animals within the herd have to be sampled, and how many isolates per faecal sample have to be tested to get a representative resistance level. For this purpose the variation in resistance among isolates within a faecal sample and the variation among faecal samples within a herd have to be investigated and taken into account.

Although several studies on prevalence of antimicrobial resistance within herds have been performed, only a few studies addressed the variation in resistance of isolates within and among faecal samples and used this information to investigate the effect of different sampling strategies on the precision of estimated resistance levels. In a study among dairy cows the variance in resistance to 12 antimicrobials was mainly attributable to variation among isolates. Based on this information four different sampling strategies were simulated and it was suggested that testing 3–4 isolates per cow (from 33 resp. 25 cows) was the best strategy to determine the prevalence of antimicrobial resistance at herd-level [17]. Among finisher pigs the largest component of variance in resistance to tetracycline and gentamicin existed between pigs within the same pen (97.5%). Bootstrap analysis based on these data revealed that for tetracycline at least 5 isolates per faecal sample need to be tested, whereas for gentamicin testing more than 10 isolates per faecal sample would result in precise estimates of resistance at herd-level [18]. Among broiler chickens the variation of resistance among isolates was low meaning that focus should be on the number of animals sampled within a flock rather than on the number of isolates tested within one animal [19]. These studies show that the composition of the variance influences the choice for a sampling strategy in order to measure antimicrobial resistance at herd-level. Due to species-specific factors that might play a role in the development and spread of antimicrobial resistant isolates within a herd (e.g. age, housing and management related factors) conclusions from these studies may not be representative for the situation within veal calf herds.

The aim of this study was to determine a sampling strategy based on the variation in proportions of resistant isolates within and among faecal samples from veal calves in order to estimate the prevalence of resistance on herd-level. For this purpose we quantified and compared the proportions of resistant commensal *E. coli* isolated from veal calves, using two different test methods (replica plating versus broth microdilution) and two different sample types (individual samples versus pooled samples).

## Materials and Methods

### Ethics Statement

The veal calf farms in this study belonged to a veal calf integration and were visited in accompany of a veterinary staff member of that integration. On the farms, selected veal calves were restrained individually and only during the time needed to obtain faecal samples. Rectal samples were taken manually using disposable gloves and a lubricant. Before this study started an animal welfare officer of the faculty of veterinary medicine, Utrecht University, was contacted and information on required permits or approvals for this study was inquired. It seemed that this study fell into the scope of the policy of the faculty on client-related animal research as the study was performed under the authority of the integration (the owner of the veal calves) and therefore no approval was required.

### Collection of Faecal Samples

Five veal calf farms were selected out of the farm database of a veal producing company based on their stage in the fattening period (e.g. end of fattening period/close to slaughter). The five selected farms cover the range in farm sizes and were considered representative for the veal calf fattening sector in The Netherlands. Some farm characteristics of the five selected farms, i.e. farm size, number of barns on the farms and administered oral group medication, are presented in Table 1.

**Table 1:** Farm characteristics of the five selected veal calf farms.

	# Calves	# Barns	Oral group medication
Farm 1	416	3	Oxytetracycline-HCl, Colistin, Doxycycline
Farm 2	1030	2	Oxytetracycline-HCl, Colistin, Ampicillin
Farm 3	1335	2	Oxytetracycline-HCl, Colistin, Tylosin
Farm 4	190	1	Oxytetracycline-HCl, Colistin, Ampicillin
Farm 5	898	3	Oxytetracycline-HCl, Colistin

Distribution of farm sizes in The Netherlands (n = 2064): 53% <450 veal calves/farm, 22% 450–750 veal calves/farm, 25% >750 veal calves/farm.

All calves received at the start of the fattening period an oral group treatment of oxytetracycline-HCl in combination with colistin. On 4 of the 5 farms an additional oral group treatment of ampicillin, doxycycline or tylosin was administered to the calves during the fattening period.

The five selected farms were all visited on the same day to collect faecal samples. On each farm, the barn closest to the entrance was selected for sampling. Within this barn five pens and two calves per pen were selected at convenience. The five pens were equally distributed over the barn and each pen housed 5–10 veal calves. The ten selected calves did not show any signs of diarrhoea or coughing and where not under antimicrobial treatment at the moment of sampling. Faecal samples were collected manually per rectum and suspended 1∶10 (w/v) in buffered peptone broth containing 30% glycerol within 24 hours. Moreover for each farm a pooled sample was made by mixing 1 ml of every individual faecal suspension. All samples were stored at −20°C until further processing.

### Susceptibility Testing and Microbiological Analysis of Faecal Samples

The stored individual and pooled faecal samples were thawed and tenfold dilution series were made in saline. From each dilution two MacConkey No.3 (MAC) agar plates (bioTRADING Benelux B.V., Mijdrecht, The Netherlands) were inoculated, each with 50 µl suspension, and incubated overnight at 37°C. From the dilution which provided up to 300 solitary colonies, one MAC agar plate was used for replica plating, and the other MAC agar plate was used for the broth microdilution test method.

### Replica Plating

Per individual or pooled sample 90 colonies with the morphological appearance of *E. coli* were selected from the MAC agar plate. The colonies were picked from the agar using sterilized toothpicks and each colony was suspended in an individual well of a 96-wells microtitre plate, resulting in 90 isolates. Each well contained 100 µl cation-adjusted Mueller Hinton broth (CAMHB) (Trek Diagnostic Systems, Basingstoke, UK) and isolates were suspended to an optical density of approximately 1–2 McFarland. Also reference strains *E. coli* ATCC 25922, *E. faecalis* ATCC 29212, *S. aureus* ATCC 29213, and *Pseudomonas aeruginosa* ATCC 27853 and two negative controls were included in each 96-wells plate to verify the antimicrobial concentration in the agar plates and reproducibility. Mueller Hinton (MH) agar plates with and without an antimicrobial breakpoint concentration were prepared and manually poured into rectangular Petri dishes (40 ml) (VWR International B.V., Amsterdam, The Netherlands). The MH agar plates contained the following antimicrobials and their breakpoint concentrations: 25 mg/L amoxicillin, 25 mg/L tetracycline, 0.5 mg/L cefotaxime, 0.125 mg/L ciprofloxacin and 8/152 mg/L tmp/s [12], [20], [21], [22]. These antimicrobials were chosen as representatives of classes of antimicrobials regularly used in veal calf production. The used breakpoint concentrations for each antimicrobial differed one or two-steps up in dilution from the EUCAST epidemiological cut-off values to prevent an overestimation of the prevalence of resistance due to an inoculum effect [23]. The concentrations in the MH agar plates were validated by inoculating isolates with known MIC values from the strain collection of CVI Lelystad. A sterile 96-pin replicator (Genetix Ltd., Hampshire, UK) was used to transfer the suspended isolates from the 96-wells plate onto a series of MH agar plates in the following order: MH containing resp. amoxicillin, tetracycline, cefotaxime, ciprofloxacin and tmp/s. MH agar plates without an antimicrobial agent were included at the beginning and end of each replicating series to control for growth [24]. Replicating was performed using one replicator per replicating series. Incubation of inoculated MH agar plates was done at 37°C for 18–20 hours. The isolates that grew on the MH agar plates containing an antimicrobial were considered to be resistant.

For two individual faecal samples (from farm 4 and 5) insufficient growth of *E. coli* was observed on the MAC agar plates to test the isolates by replica plating (<90 colonies). Therefore these samples were excluded from the replica plating dataset.

In total 4320 isolates from 48 individual samples and 450 isolates from 5 pooled samples were tested for their resistance against the five antimicrobials by replica plating.

### Broth Microdilution

From the second MAC agar plate ten colonies with the morphological appearance of *E. coli* were selected per individual and pooled sample and confirmed as *E. coli* by a positive indole test. For these ten *E. coli* isolates per sample Minimum Inhibitory Concentrations (MIC) were determined by broth microdilution according to ISO standard 20776-1-2006. Briefly, from fresh overnight cultures on blood agar, inocula were prepared in saline with a turbidity of 0.5 McFarland. Final inocula were prepared by 200-fold dilution of these suspensions in CAMHB. Microtitre trays (EUMVS type, Trek Diagnostic Systems, Basingstoke, UK) with dehydrated dilution ranges of panels of antibiotics were used to monitor resistance to ampicillin, tetracycline, cefotaxime, ciprofloxacin and tmp/s. Each well of the microtitre tray was inoculated with 50 µl of the final inoculum. ATCC strains *E. coli* 25922 and *E. faecalis* 29212 were included to monitor the quality of the results. After inoculation of the wells the microtitre trays were sealed and incubated at 35°C for 18 to 24 hours. EUCAST epidemiological cut-off values (ampicillin ≤8 mg/L, tetracycline ≤8 mg/L, cefotaxime ≤0.25 mg/L, ciprofloxacin ≤0.03 mg/L, trimethoprim ≤2 mg/L, and sulphamethoxazole ≤64 mg/L) were used to differentiate between wild-type isolates and isolates with reduced susceptibility [25]. Isolates with non-wild type susceptibility to both trimethoprim and sulphamethoxazole were considered resistant to tmp/s.

In total 500 isolates from 50 individual samples and 50 isolates from 5 pooled samples were tested for their resistance using broth microdilution.

### Statistical Analysis

#### Logistic regression analysis

A generalised linear mixed model with a logit link function was used to study the relationship between the binomial distribution of the grouped outcome (number of resistant isolates on the total number of isolates tested per faecal sample) and explanatory variables antimicrobial, test method and sample type, including the 2-way interaction between antimicrobial and test method or sample type and the 3-way interaction between antimicrobial, test method and sample type. A random farm, a random pen and a random faecal sample effect were included in the model to take into account the clustering of faecal samples within farms and pens, and the clustering of isolates within a faecal sample.

The random effects were assumed to have a compound symmetry covariance structure. A backward stepwise selection on the full model was performed to find the best fitting model to describe the dataset. Selection of the best fitting model was based on the value of Akaike’s Information Criteria (AIC). The model with the lowest AIC value was considered the best fitting model, with the AIC being the −2*loglikelihood +2*the number of parameters in the model. The odds ratio and 95% confidence interval for the explanatory variables in the best fitting model were calculated.

The estimated variance values of the random effects in the best fitting model were used to examine the variation (proportion from total) in antimicrobial resistance on farm-level, pen-level and sample-level, with the variance of a standard logistic density fixed at ð^2^/3 [26]. The analyses were performed using open-source programme R, version 2.12.2 [27] and package lme4, version 0.999375-42 [28] for generalized linear mixed-effects models using the Laplace approximation method.

#### Bootstrap analysis

Bootstrap analysis was performed to test possible sampling strategies for their effect on estimated proportions of resistance within a sample, based on the distribution of data in the observed dataset [29]. The analysis was based on the dataset containing the resistance results of 4320 isolates collected from the individual veal calf samples and tested by replica plating. This dataset was used because replica plating is our preferred test method based on precision, feasibility and cost effectiveness. Also the dataset contained more data and obtained similar results compared to the broth microdilution dataset (Table 2).

**Table 2:** Logistic regression analysis of explanatory variables test method and sample type including their interaction with the tested antimicrobials on the odds of resistant isolates in faecal samples.

Variable		*b*	SE(*b*)	*p*	OR	95% CI	
						Lower	Upper
Test method	Broth microdilution	Reference			1		
	Replica plating - Amox	0.20	0.11	0.07	1.23	0.98	1.53
	Replica plating - Cip	−1.23	0.14	<2e-16	0.29	0.22	0.38
	Replica plating - Tet	0.23	0.18	0.19	1.26	0.89	1.79
	Replica plating - Tmp/s	0.12	0.11	0.31	1.12	0.90	1.40
Sample type	Individual sample	Reference			1		
	Pooled sample - Amox	−0.56	0.62	0.36	0.57	0.17	1.91
	Pooled sample - Cip	−0.65	0.64	0.31	0.52	0.15	1.84
	Pooled sample - Tet	0.19	0.64	0.77	1.21	0.34	4.27
	Pooled sample - Tmp/s	−0.26	0.62	0.68	0.77	0.23	2.59

Amox = amoxicilline, Cip = ciprofloxacine, Tet = tetracycline, Tmp/s = trimethoprim/sulfa-methoxazole.

Variance random sample effect = 1.561.

Variance random pen effect = 0.084.

Variance random farm effect = 1.413.

Eight sampling strategies, each representing a different composition of a pooled sample, were simulated. These strategies were based on the variability of resistance within and between samples and included various compositions of pooled samples varying the number of isolates and number of samples to investigate the precision of the sampling strategies on the estimated prevalence of resistance. The simulation of a pooled sample was justified, because logistic regression analysis showed that the odds of an isolate being resistant did not significantly differ between individual and pooled samples (Table 2).

The sampling strategies, in part based on Dunlop et al. [18], lead to total sample sizes of 20, 40 and 100 isolates tested per farm, representing a small, medium or large sample size from a practical point of view. The various compositions of a pooled sample were implemented to determine if focus should be on the number of faecal samples included or on the number of isolates tested per sample in order to get precise farm resistance levels.

Per sampling strategy and for each antimicrobial, a set of 1000 bootstrap samples was drawn from the dataset (individual samples tested with replica plating) with replacement, where each observation had an equal probability of selection. Within each bootstrap sample the sampling strategy was simulated per farm by selecting, with replacement, first the number of faecal samples (varying from 4–20 faecal samples depending on the sampling strategy) and secondly within these faecal samples the number of isolates (varying from 2–20 isolates per sample depending on the sampling strategy). Each bootstrap sample comprised the data of the simulated sampling strategy from all five farms combined.

Logistic regression analysis for grouped data (pooled sample per farm) and logit link was performed on each bootstrap sample, and the estimated coefficient for the intercept was used to calculate the proportion isolates resistant to each antimicrobial (proportion resistant isolates = e ^intercept/^(1+ e ^intercept^)). Based on the distribution of these estimated proportions from 1000 bootstrap samples per sampling strategy, values for the mean, standard deviation and the 2.5th and 97.5th percentile were calculated. Bootstrap analysis was performed using open-source programme R, version 2.12.2 [27].

## Results

### Descriptive Statistics

The number and proportion of isolates tested resistant for each antimicrobial by replica plating and broth microdilution using individual and pooled samples are presented in Table 3. The MIC distribution of isolates tested with broth microdilution is presented in Table 4. The proportion of resistant isolates tested among individual samples by replica plating was high for tetracycline (0.92), intermediate for amoxicillin and tmp/s (0.61 and 0.47, respectively) and low for ciprofloxacin and cefotaxime (0.11 and 0.02, respectively). Due to the lack of resistant isolates for cefotaxime in broth microdilution the results for cefotaxime were left out the statistical model.

**Table 3:** Proportions and numbers of *E. coli* isolates tested resistant by replica plating and broth microdilution in individual and pooled samples.

	Individual samples		Pooled samples	
	RP	BMD	RP	BMD
Antimicrobial	(n = 4320)	(n = 500)	(n = 450)	(n = 50)
Amoxicillin	0.61 (n = 2652)	0.57 (n = 287)	0.50 (n = 225)	0.54 (n = 27)
Cefotaxime	0.02 (n = 83)	0 (n = 0)	0.004 (n = 2)	0 (n = 0)
Ciprofloxacin	0.11 (n = 475)	0.23 (n = 114)	0.06 (n = 28)	0.14 (n = 7)
Tetracycline	0.92 (n = 3963)	0.91 (n = 453)	0.95 (n = 427)	0.92 (n = 46)
Trimethoprim - sulfamethoxazole	0.47 (n = 2015)	0.44 (n = 220)	0.40 (n = 182)	0.44 (n = 22)

RP = Replica plating test method. BMD = Broth microdilution test method.

**Table 4:** MIC distribution of 500 *E. coli* isolates (in %) tested for their antimicrobial susceptibility to ampicillin, tetracycline, cefotaxime, ciprofloxacin, trimethoprim and sulphamethoxazole by broth microdilution.

E. coli	MIC distribution mg/L (%)																		
N = 500	0	0.015	0.03	0.06	0.125	0.25	0.5	1	2	4	8	16	32	64	128	256	512	1024	2048
Ampicillin							0	0.2	8.2	30.6	3.6	0	0	57.4					
Tetracycline								0.2	3.6	5.2	0.4	0	0.2	10.4	80				
Cefotaxime				58.8	37.8	3.4	0	0											
Ciprofloxacin	0.4	34	42.8	7.8	4.2	9	0.8	0	0	0	0.4	0.6							
Trimethoprim							50.6	2.6	0.2	0	0	0	1.4	45.2					
Sulfamethoxazole											45.4	1	0	0	0.2	0	0	0.8	52.6

### Logistic Regression Analysis

Based on the AIC, the best fitting model included explanatory variables antimicrobial, test method and sample type and the 2-way interaction term between antimicrobial and test method and between antimicrobial and sample type. The 3-way interaction term between antimicrobial, test method and sample type was left out of the model because it was not significant.

The best fitting model showed no significant difference in the odds of resistant isolates using replica plating compared to broth microdilution for the antimicrobials amoxicillin (OR 1.23, *p* = 0.07), tetracycline (OR 1.26, *p* = 0.19) and tmp/s (OR 1.12, *p* = 0.31) (Table 2). For ciprofloxacin, on the other hand, a significant lower odds of resistant isolates was found by replica plating (OR 0.29, *p* = <2e-16 ). Pooled samples showed lower odds of resistant isolates for the antimicrobials amoxicillin, ciprofloxacin and tmp/s (OR<1) and a higher odds of resistant isolates for tetracycline (OR = 1.21) compared to individual samples, although these differences were not significant (p>0.05).

Based on the estimated variance values of the random effects in the best fitting model, the largest proportion of total variance was found between faecal samples, followed by variance between farms and pens (0.25, 0.22 and 0.01 resp.).

### Bootstrap Analysis

The effect of sampling strategy on the distribution of the estimated proportion of *E. coli* isolates resistant to the five tested antimicrobials is presented in Table 5. The various sampling plans yielded consistent values for the estimated mean proportion of isolates resistant to amoxicillin (0.61) tetracycline (0.92), ciprofloxacin (0.11) and tmp/s (0.46–0.47).

**Table 5:** Proportion *E. coli* isolates resistant to amoxicillin (25 mg/L), tetracycline (25 mg/L), ciprofloxacin (0.125 mg/L) and trimethoprim/sulfamethoxazole (8/152 mg/L) obtained with 8 different sampling strategies.

						Precision
		# Isolates	Total number			2.5^th^–97.5^th^
Strategy	# Samples	per sample	of isolates	Mean	S.D.	Percentiles
Proportion of *E. coli* resistant to 25 mg/L amoxicillin						
1	4	5	20	0.61	0.06	0.50–0.73
2	5	4	20	0.61	0.06	0.51–0.72
3	10	2	20	0.61	0.05	0.52–0.70
4	10	4	40	0.61	0.04	0.54–0.69
5	20	2	40	0.61	0.03	0.55–0.68
6	5	20	100	0.61	0.04	0.53–0.70
7	10	10	100	0.61	0.03	0.55–0.68
8	20	5	100	0.61	0.03	0.56–0.66
Proportion of *E. coli* resistant to 25 mg/L tetracycline						
1	4	5	20	0.92	0.04	0.82–0.98
2	5	4	20	0.92	0.04	0.83–0.98
3	10	2	20	0.92	0.03	0.85–0.98
4	10	4	40	0.92	0.03	0.86–0.96
5	20	2	40	0.92	0.02	0.87–0.96
6	5	20	100	0.92	0.03	0.85–0.97
7	10	10	100	0.92	0.02	0.87–0.96
8	20	5	100	0.92	0.03	0.85–0.97
Proportion of *E. coli* resistant to 0.125 mg/L ciprofloxacin						
1	4	5	20	0.11	0.05	0.03–0.21
2	5	4	20	0.11	0.04	0.03–0.20
3	10	2	20	0.11	0.03	0.05–0.18
4	10	4	40	0.11	0.03	0.05–0.17
5	20	2	40	0.11	0.02	0.06–0.16
6	5	20	100	0.11	0.04	0.04–0.19
7	10	10	100	0.11	0.03	0.06–0.17
8	20	5	100	0.11	0.02	0.07–0.15
Proportion of *E. coli* resistant to 8/152 mg/L trimethoprim/sulfamethoxazole						
1	4	5	20	0.46	0.06	0.36–0.58
2	5	4	20	0.47	0.05	0.36–0.58
3	10	2	20	0.47	0.05	0.38–0.55
4	10	4	40	0.47	0.04	0.40–0.54
5	20	2	40	0.46	0.03	0.40–0.53
6	5	20	100	0.46	0.04	0.39–0.55
7	10	10	100	0.47	0.03	0.40–0.53
8	20	5	100	0.47	0.03	0.42–0.52

using bootstrap resampling.

Precision of the estimated mean proportions of each sampling strategy was determined by the width of the interval between the 2.5^th^ and 97.5^th^ percentile. The 2.5^th^ and 97.5^th^ percentile interval became more narrow when increasing the total number of isolates tested. Also within a fixed total number of isolates tested increasing the number of samples and consequently decreasing the number of isolates tested per sample resulted in a narrower precision interval. All bootstrap results were estimable and no extreme prevalence values were estimated.

## Discussion

In search for a sampling strategy to estimate resistance levels within a veal calf herd we investigated the reliability of the replica plating test method by comparing the obtained results to broth microdilution as a reference. With replica plating isolates were tested for their resistance using breakpoint concentrations one or two twofold dilution steps higher compared to EUCAST epidemiological cut-off values. Nevertheless, this method has proven to provide results comparable to those obtained with broth microdilution for amoxicillin, tetracycline and tmp/s. For ciprofloxacin a significantly lower odds of resistant isolates by replica plating was found. Two other studies also compared the prevalence of resistant isolates per sample using antimicrobial containing agar plates versus a dilution test method as reference using different breakpoints. Vieira et al. [15] compared the prevalence of tetracycline and sulphonamide resistance between both methods using similar breakpoint concentrations for tetracycline, whereas the agar plates contained a breakpoint concentration one dilution step up for sulphonamide. Österblad et al. [24] compared the prevalence of resistance to ampicillin, trimethoprim and tetracycline using breakpoint concentrations one step dilution up for ampicillin, and one step dilution down for trimethoprim and tetracycline compared to the reference method. Despite the use of different breakpoint concentrations compared to the reference method, the rate of resistance detection did not differ statistically between the test methods for any of the antibiotics tested. These results can be explained by the bimodal distribution of MIC values of *E. coli* isolates around the used breakpoint concentrations for ampicillin, tetracycline, cefotaxime and trimethoprim and sulphamethoxazole (Table 4). For this reason the use of a higher breakpoint concentration of these antimicrobials in an agar plate did not give different resistance results compared to broth microdilution using EUCAST epidemiological cut-off values. For ciprofloxacin, on the other hand, MIC values of the isolates were distributed around the breakpoint concentrations used within both test methods due to the fact that development of resistance to ciprofloxacin increases gradually by single-step mutations in the chromosome [30]. Thus, a one or two-step higher dilution in breakpoint concentration resulted in a lower prevalence of resistance to ciprofloxacin found by replica plating.

Besides test method, also the application of pooled samples was investigated in this study. Estimating the proportion of resistant isolates within a herd based on pooled faecal samples is convenient and least expensive to perform compared to individual samples. This study showed that the use of pooled samples is justified as the odds of finding resistant isolates within pooled samples did not significantly differ compared to individual samples. This is in accordance with studies among finisher pigs and feedlot cattle [18], [31], although Wagner et al. [31] only found similar resistance patterns for individual and pooled samples when prevalence of resistance to an antimicrobial was >2%. Whereas the study of Dunlop et al. [18] was based on a replica plating test method, Wagner et al. [31] used broth microdilution, but could not detect any resistant isolate within pooled samples of 10 samples for antimicrobials with a low prevalence. In this study the prevalence of isolates resistant to cefotaxime was also low and resistant isolates were not detected among individual or pooled samples when tested by broth microdilution. Surprisingly, isolates resistant to cefotaxime in individual and pooled samples were detected by replica plating even though the breakpoint concentration was one-step higher in dilution compared to broth microdilution. The fact that resistant isolates were found by replica plating is probably due to the higher number of isolates tested within each sample which increased the chance of picking at least one resistant isolate. This result emphasizes the advantage of testing multiple isolates per faecal sample for the determination of resistance, especially when resistance is rare.

Simulation studies are used to investigate the impact of number of isolates and number of samples tested per herd on the precision of the estimated prevalence of resistance. These studies are usually based on prevalence of resistance obtained from one herd or flock of one farm only [17], [18], [19]. Since resistance patterns can be clustered within a specific herd, variation between different herds can be large [32]. Therefore, this study was based on the resistance results from five different farms to take possible farm influences into account.

Composition of variance showed that variation among farms accounted for 22% of the total variance in resistance, variation among samples for 25% and variation among pens for 1%. Based on these variances eight sampling strategies were simulated to investigate the influence of several compositions of a pooled sample on the precision of the estimated prevalence of resistance on herd-level. All strategies provided comparable estimates of the mean prevalence of resistance within each antimicrobial. Small improvements in the precision of the estimated mean were obtained by testing a larger total number of isolates and testing more faecal samples relative to the number of isolates within a faecal sample. It is not surprising that testing more isolates per sample will result in a more precise estimate of the mean. More interesting is the fact that the precision interval is more influenced by the number of faecal samples included in a pooled sample. An explanation for this is the fact that the largest proportion of total variation was found between samples. This is in accordance to a study of Dunlop among finisher pigs where 97,5% of the total variance was attributable to variance between individual pigs [18].

The aim of this study was to determine one optimal sampling strategy to estimate the prevalence of resistance to amoxicillin, tetracycline, cefotaxime, ciprofloxacin and tmp/s within veal calf herds. In our study, the optimal sampling strategy from the perspective of precision of the estimated levels of resistance and practicality, consists of a pooled faecal sample from 20 individual animals, from which 90 isolates are tested for their susceptibility by replica plating.
