# Correction: Quantifying Antimicrobial Resistance at Veal Calf Farms

**DOI:** 10.1371/annotation/f9a8e916-bef3-466c-b45f-a1f71a7e8213

**Published:** 2013-01-02

**Authors:** Angela B. Bosman, Jaap Wagenaar, Arjan Stegeman, Hans Vernooij, Dik Mevius

The second author's name was spelled incorrectly. The correct name is: Jaap A. Wagenaar.

The correct citation is: Bosman AB, Wagenaar JA, Stegeman A, Vernooij H, Mevius D (2012) Quantifying Antimicrobial Resistance at Veal Calf Farms. PLoS ONE 7(9): e44831. doi:10.1371/journal.pone.0044831

There is also an error in the Materials and Methods section. In Statistical Analysis, the first sentence of the third paragraph of "Logistic regression analysis" should read: The estimated variance values of the random effects in the best fitting model were used to examine the variation (proportion from total) in antimicrobial resistance on farm-level, pen-level and sample-level, with the variance of a standard logistic density fixed at π2/3 [26]. 

